# Cisplatin-induced ERK1/2 activity promotes G1 to S phase progression which leads to chemoresistance of ovarian cancer cells

**DOI:** 10.18632/oncotarget.24884

**Published:** 2018-04-13

**Authors:** Michal Kielbik, Damian Krzyzanowski, Bartlomiej Pawlik, Magdalena Klink

**Affiliations:** ^1^ Institute of Medical Biology, Polish Academy of Sciences, Lodz, Poland; ^2^ Institute of Microbiology, Biotechnology and Immunology, University of Lodz, Lodz, Poland

**Keywords:** ovarian cancer cells, cisplatin resistance, ERK1/2 signaling pathway, cell cycle, MEK1/2 inhibitor

## Abstract

The link between ERK1/2 activity and cisplatin cytotoxicity, in association with the cell cycle, in ovarian cancer cell lines resistant (A2780cis; SK-OV-3) and sensitive (A2780) to cisplatin was determined. We observed that cisplatin, at a low concentration enhanced the activation of ERK1/2 in A2780 cells and increased their accumulation in the S phase, resulting in low cytotoxicity. A high concentration of drug induced dephosphorylation and degradation of ERK1/2 and was extremely toxic, accumulating most of to these cells in the sub-G1 phase. The PD98059, pharmacological inhibitor of ERK1/2 activation, increased the cytotoxicity of cisplatin applied at a low concentration to A2780 cells (decreased ERK1/2 activity), causing shift of cell accumulation from the S to G1 phase. Surprisingly, PD98059 enhanced cell viability when a chemotherapeutic was used at high concentration, intensifying phosphorylation level of ERK1/2 and reversing cell cycle arrest in sub-G1 to promote the G1 and S phases. A2780cis cells demonstrated resistance to cisplatin with high ERK1/2 activity and accumulation of cells in the G1 and S phases. PD98059 sensitized resistant cells to drug toxicity during the first 24 hours of treatment, with blocked ERK1/2 phosphorylation and prevented progression from the G1 to S phase. SK-OV-3 resistant cells characterized with extremely high basal phosphorylation of ERK1/2, which wasn't changed after exposure to cisplatin. Administration of PD98059 didn't change the cytotoxicity of cisplatin in these cells. In conclusion, ERK1/2, activated by cisplatin, participates in the cell cycle progression from the G1 to S phase, enhancing cells’ survival and drug resistance.

## INTRODUCTION

Ovarian cancer cells are primarily sensitive to chemotherapy based on platinum compounds and taxanes with an initial response of patients that is high in up to 70%-80% of cases. However, the majority of patients, up to 70% in the advanced stage of disease, suffer from disease relapse within 6-24 month after treatment. Most of the relapse patients have acquired platinum resistance. Initially, sensitive cancer cells become resistant with repeated chemotherapy cycles. Moreover, the high heterogeneity of ovarian cancer cells in the tumor microenvironment is also responsible for the expansion of primary platinum-resistant cells. Therefore, recurrent ovarian cancer is rather incurable, and the 5-year survival rate of patients with the advanced stage of disease is only approximately 20%-35% [1, 2].

Cisplatin activity is mainly connected to its interaction with DNA. This drug forms covalent bonds with the N^7^ position of purine bases to create intra-strand and inter-strand crosslinks that cause conformational changes, blocking DNA replication and transcription and inducing cell death. There are other proposed mechanisms of cisplatin cytotoxicity, such as interactions with mitochondrial DNA, leading to mitochondrial dysfunction, disruption of calcium homeostasis or generation of oxidative stress, which can also lead to apoptotic or necrotic cell death. It is worth noting that due to cisplatin-induced genotoxic stress, multiple signaling pathways that may contribute to apoptosis or to the development of chemoresistance are activated. Among others, protein kinase C, mitogen-activated protein kinases and the PI3-K/AKT pathway seem to play critical roles in the cisplatin effect on cancer cell function [3–6].

Mitogen-activated protein kinases (MAPKs) belong to a family of serine/threonine kinases mediating signal transduction in a response to various stimuli, such as growth factors, cytokines, mitogens, and oxidative stress. MAPKs have been classified into a few subfamilies in which pathways based on extracellular signal-regulated kinases 1 and 2 (ERK1/2) are included. Like all MAPKs, ERK1/2 undergo activation through a signaling cascade in which Ras protein, Raf kinase, and mitogen activated protein kinase kinase 1 and 2 (MEK1/2) are sequentially involved. In normal, physiological conditions, ERK1/2 promotes cell differentiation and proliferation as well as regulates the cell cycle and apoptosis [7–9]. However, this role can be altered in pathological conditions, such as tumor. Enhanced/constitutive activation of ERK1/2 occurs in various human cancers, which is related to mutations in genes encoding Ras and B-Raf proteins and/or due to the oxidative stress observed in the tumor microenvironment. Elevated activation of the Ras/Raf/ERK pathway is commonly thought to have anti-apoptotic and chemoresistant effects that promote cancer cell survival and tumor progression. Conversely, this pathway also appears to have the opposite role in which over activation of this pathway leads to cell cycle arrest and provides pro-apoptotic signals [7, 10]. Moreover, therapeutic drugs (platinum compounds and paclitaxel) have been shown to activate ERK1/2 in several tumor cell types, including ovarian cancer cells [3, 11, 12]. Nevertheless, controversies remain whether cisplatin-induced ERK1/2 activation contributes to ovarian cancer cell survival or death.

In these studies, we sought to determine the role and possible mechanism of ERK1/2 action in the response of ovarian cancer cells to treatment with cisplatin. We used two matched cell lines, one sensitive (A2780) and one with developed cisplatin resistance (A2780cis), as well as one additional cell line with proved cisplatin resistance - SK-OV-3.

## RESULTS

### Cisplatin-induced ERK1/2 activation effect on ovarian cancer cell line viability

First, we measured the half maximal effective concentration (EC_50_) values for cisplatin after 48 hours of cell culture using the MTT test. There was 3-fold higher sensitivity with A2780 cells (EC_50_ =10.33 μM) than with A2780cis cells (EC_50_ =32.06 μM) and almost 4-fold higher than with SK-OV-3 (38.06 μM).

**Figure 1:**
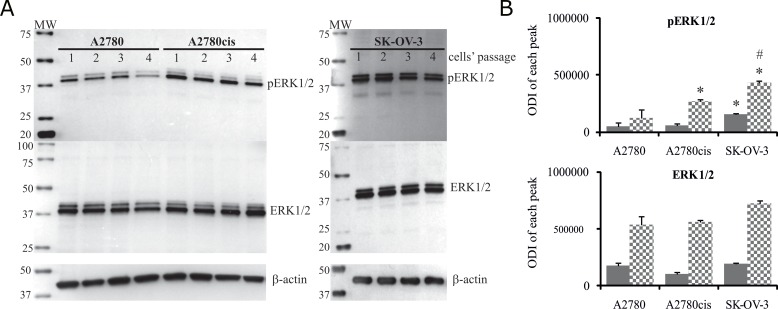
Total level of ERK1/2 and their phosphorylation states in A2780, A2780cis and SK-OV-3 cells Cultured cells were harvested and lysed, and the levels of total and phosphorylated ERK1/2 proteins were determined. **(A)** Immunoblots from four independent passages are presented. **(B)** Bands were quantified by densitometric analysis and data are presented as the optical density intensity (ODI) of the area under each band’s peak ± SD. ERK1 – gray column and ERK2 - checkered column. Statistical significance: ^*^A2780cis and SK-OV3 vs. A2780, p=0.002 (unpaired *t*-test); ^#^SK-OV-3 vs. A2780cis, p=0.01 (unpaired *t*-test).

**Figure 2:**
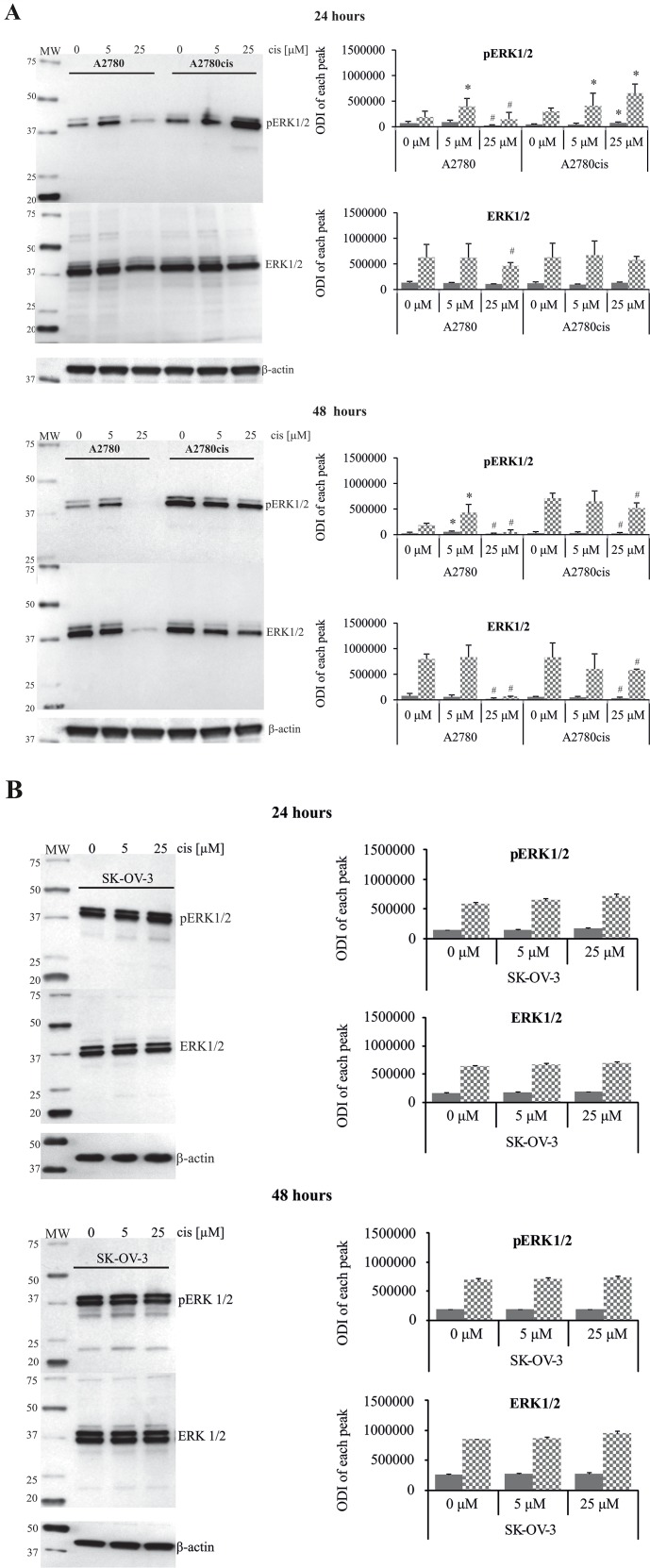
Effect of cisplatin on the total amount and phosphorylation state of ERK1/2 in A2780, A2780cis and SK-OV-3 cells A2780 and A2780cis **(A)**, as well as SK-OV-3 **(B)** cells were treated with cisplatin at concentrations of 5 μM or 25 μM for 24 or 48 hours or left untreated. Representative immunoblots of the ERK1/2 protein level and their phosphorylation are presented. Bands were quantified by densitometric analysis and data are presented as the optical density intensity (ODI) of the area under each band’s peak ± SD from 5 independent experiments. ERK1 – gray column and ERK2 - checkered column. ^*^Statistically significant increase: cisplatin treated cells vs. untreated cells, p=0.043; ^#^Statistically significant decrease: cisplatin treated cells vs. untreated cells, p=0.043 (Wilcoxon’s signed-rank test).

**Figure 3:**
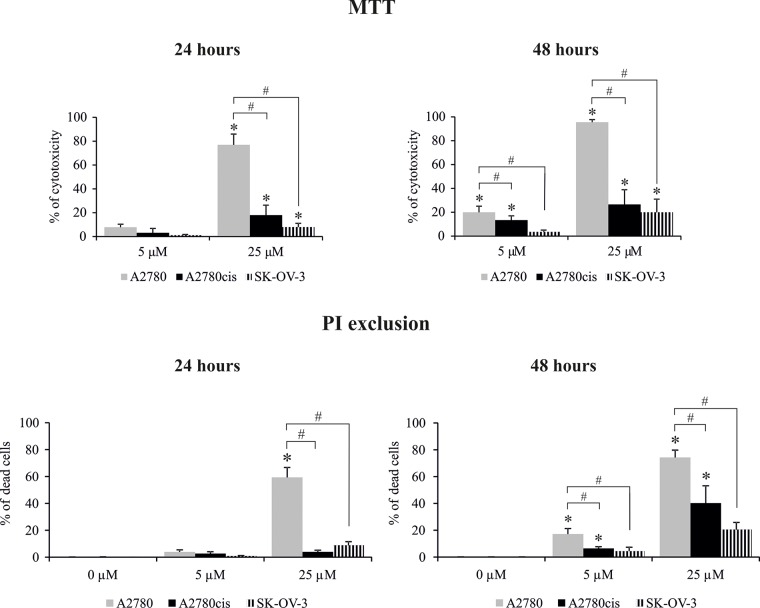
Effect of cisplatin on the viability of A2780, A2780cis and SK-OV-3 cells Cells were treated with cisplatin at concentrations of 5 μM or 25 μM for 24 or 48 hours or they were left untreated. The viability of cells was determined with the MTT assay and data are presented as the percentage of cytotoxicity ± SD from 6 independent experiments or with PI exclusion assay, and data are presented as percentage of dead cells ± SD from 4 independent experiments. Statistical significance: ^*^cisplatin treated cells vs. untreated cells, p≤0.01 (paired *t*-test). ^#^A2780 vs. A2780cis and SK-OV-3 cells, p≤0.01 (unpaired *t*-test).

Next, we analyzed the time kinetics of ERK 1 and 2 activation, measured as a phosphorylation state, in cancer cell lines treated with 5 μM and 25 μM of cisplatin for various time periods. In parallel, we tested the viability of these cells based on the MTT test and propidium iodide (PI) exclusion assay to determine the possible role of ERK1/2 in ovarian cancer cell resistance to cisplatin. As demonstrated in Figure 1, cancer cell lines had constitutively phosphorylated ERK1/2; however, cisplatin resistant cells A2780cis and SK-OV-3 had 2.1- and 3.4-fold higher phosphorylation of ERK1/2 proteins than sensitive A2780 cells. Moreover, resistant SK-OV-3 cells had 2-fold higher phosphorylation of signaling proteins than resistant A2780cis cells. The total level of these kinases between paired A2780 and A2780cis cell lines did not vary but was lower than in SK-OV-3 cells. Treatment of cancer cell lines with cisplatin for 0.5, 1, 3 or 6 hours had no effect on the initial level or phosphorylation of ERK1/2 or on the cell survival (data not shown). Changes in the activity of ERK1/2 (Figure 2A and 2B) and cell viability (Figure 3) were observed after 24 and 48 hours of culturing ovarian cancer cell lines with cisplatin. The degree of kinases phosphorylation were trace in A2780 cells exposed to cisplatin at a concentration of 25 μM after 24 or 48 hours of treatment. The basal levels of proteins were slightly (24 hours) or highly (48 hours) reduced. Simultaneously, the cytotoxicity of drug reached 59 or 76% and 74 or 95%, for PI and MTT tests, respectively. In contrast, 5 μM cisplatin significantly enhanced the phosphorylation of both kinases and cytotoxicity of the drug was only at the level of 3 or 7% (insignificant) and 17 or 20% for PI and MTT test, respectively after 24 and 48 hours of cell exposure. Cisplatin at low and high concentrations significantly induced ERK1/2 phosphorylation in resistant A2780cis cells exposed to this drug for 24 hours without changing their baseline levels. However, stronger phosphorylation of ERK1/2 was caused by 25 μM of cisplatin. It is worth mentioning that the percentages of dead cells did not exceed 2 or 3 and 4 or 18 for PI and MTT tests, respectively, and the effect of 5 μM of cisplatin was statistically insignificant. Prolongation of the exposure time (48 hours) of A2780cis cells to cisplatin at both concentrations slightly decreased the protein level, although the kinases remained highly phosphorylated. ERK1/2 activity was accompanied by relatively low cytotoxicity of the chemotherapeutic, which was 6 or 13% and 26 or 40% for 5 and 25 μM, depending on the cytotoxicity test used. The treatment of SK-OV-3 cells with cisplatin at both concentrations did not affect the phosphorylation of kinases, independently of culture time. The significant cytotoxicity was observed only at concentration of 25 μM. Both MTT and PI exclusion tests showed similar cytotoxicity at the level of 8% and 20% during 24 and 48 hours of culturing. These data indicate that SK-OV-3 cells are resistant to cisplatin at similar rate as A2780cis cells.

### Effect of MEK1/2 inhibitor on the sensitivity of ovarian cancer cells to cisplatin

**Figure 4:**
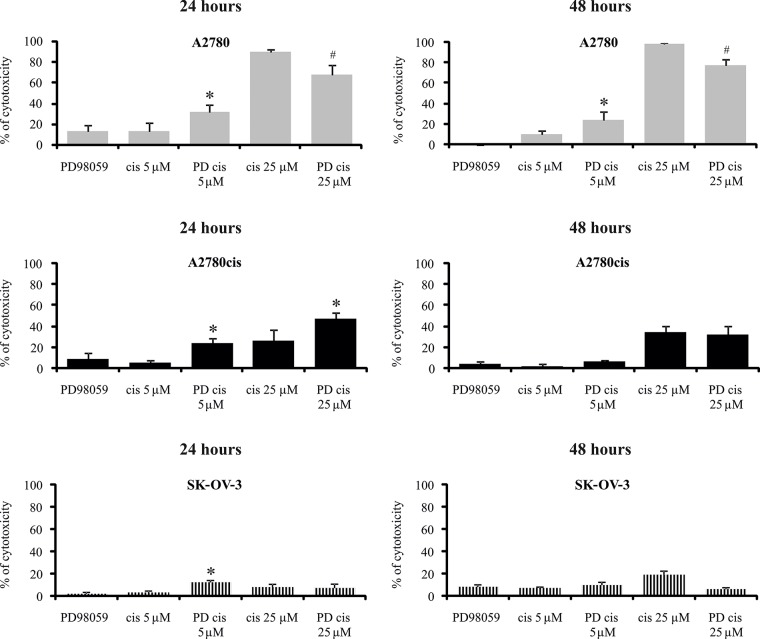
Effect of MEK1/2 inhibitor on the sensitivity of ovarian cancer cells to the cytotoxicity of cisplatin Cells were first treated with 50 μM of PD98059 for 1 hour and then cultured with cisplatin at concentrations of 5 μM or 25 μM for 24 or 48 hours or left untreated. The viability of cells was determined with the MTT assay and data are presented as the percentage of cytotoxicity ± SD from 6 independent experiments. ^*^Statistically significant increase: cisplatin + PD98059 vs. cisplatin, p≤0.002; ^#^Statistically significant decrease: cisplatin + PD98059 vs. cisplatin, p≤0.002 (paired *t*-test).

The possible role of ERK1/2 in regulating cisplatin cytotoxicity against resistant and sensitive cells was also evaluated using a pharmacological inhibitor of upstream kinases MEK1/2 – PD98059. First, we chose an efficient concentration of PD98059 that inhibited ERK1/2 activation. MEK1/2 inhibitor, at a concentration of 50 μM, effectively blocked basal phosphorylation of ERK1/2 in tested cell lines and demonstrated acceptable cytotoxicity (Supplementary Figure 1); therefore, this concentration was used for the subsequent experiments. It is noteworthy that the action of MEK1/2 inhibitor on the cisplatin-induced cell death strongly depended on the cell type, therapeutic drug concentration and treatment time (Figure 4). As expected, PD98059 more than two-fold enhanced the cytotoxic activity of cisplatin applied at a low concentration against the A2780 cell line after 24 and 48 hours. This shows that active ERK1/2 participates in keeping cells alive in the presence of cisplatin applied at a low concentration. In contrast, PD98059 surprisingly abrogated the cytotoxicity of 25 μM cisplatin against sensitive cells during 24 or 48 hours of treatment, although it should be noted that most A2780 cells remained dead. Inhibition of ERK1/2 activity resulted in greater cytotoxicity of cisplatin, at both concentrations, against the A2780cis cells after 24 hours of treatment. These data indicate that ERK1/2 are important proteins for A2780cis cells to resist cisplatin cytotoxicity during short time of treatment. However, prolongation of the culture time up to 48 hours diminished the effect of MEK1/2 inhibitor on the survival of A2780cis cells treated with the tested drug. PD98059 caused that non-toxic 5 μM concentration of cisplatin became slightly toxic and induced cell death at 12% during 24 hours of culture SK-OV-3 cells. However, this inhibitor did not change the cytotoxicity of 25 μM cisplatin against these cells. Similarly to A2780cis cells, PD98059 did not influence SK-OV-3 cells’ survival during 48 hours of culture with cisplatin at both concentrations (Figure 4).

**Figure 5:**
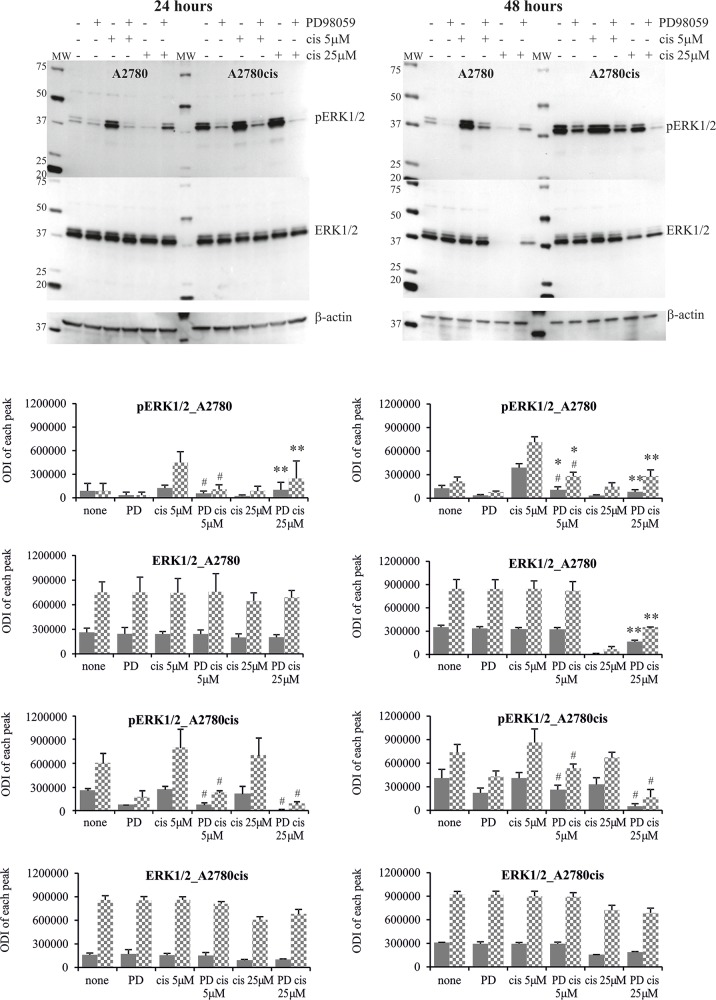
Phosphorylation of ERK1/2 in A2780 and A2780cis cells treated with MEK1/2 inhibitor and cisplatin Cells were first treated with 50 μM PD98059 for 1 hour and then cultured with cisplatin at the concentrations of 5 μM or 25 μM for 24 or 48 hours or left untreated. Representative immunoblots of the ERK1/2 protein level and their phosphorylation are presented. Bands were quantified by densitometric analysis and data are presented as the optical density intensity (ODI) of the area under each band’s peak ± SD from 5 independent experiments. ERK1 – gray column and ERK2 - checkered column. Statistically significant decrease: ^#^cisplatin + PD98059 vs. cisplatin, p≤0.05; Statistically significant increase: ^*^cisplatin + PD98059 vs. PD9858, p≤0.05; ^**^cisplatin + PD98059 vs. cisplatin, p≤0.05 (Wilcoxon’s signed-rank test).

**Figure 6:**
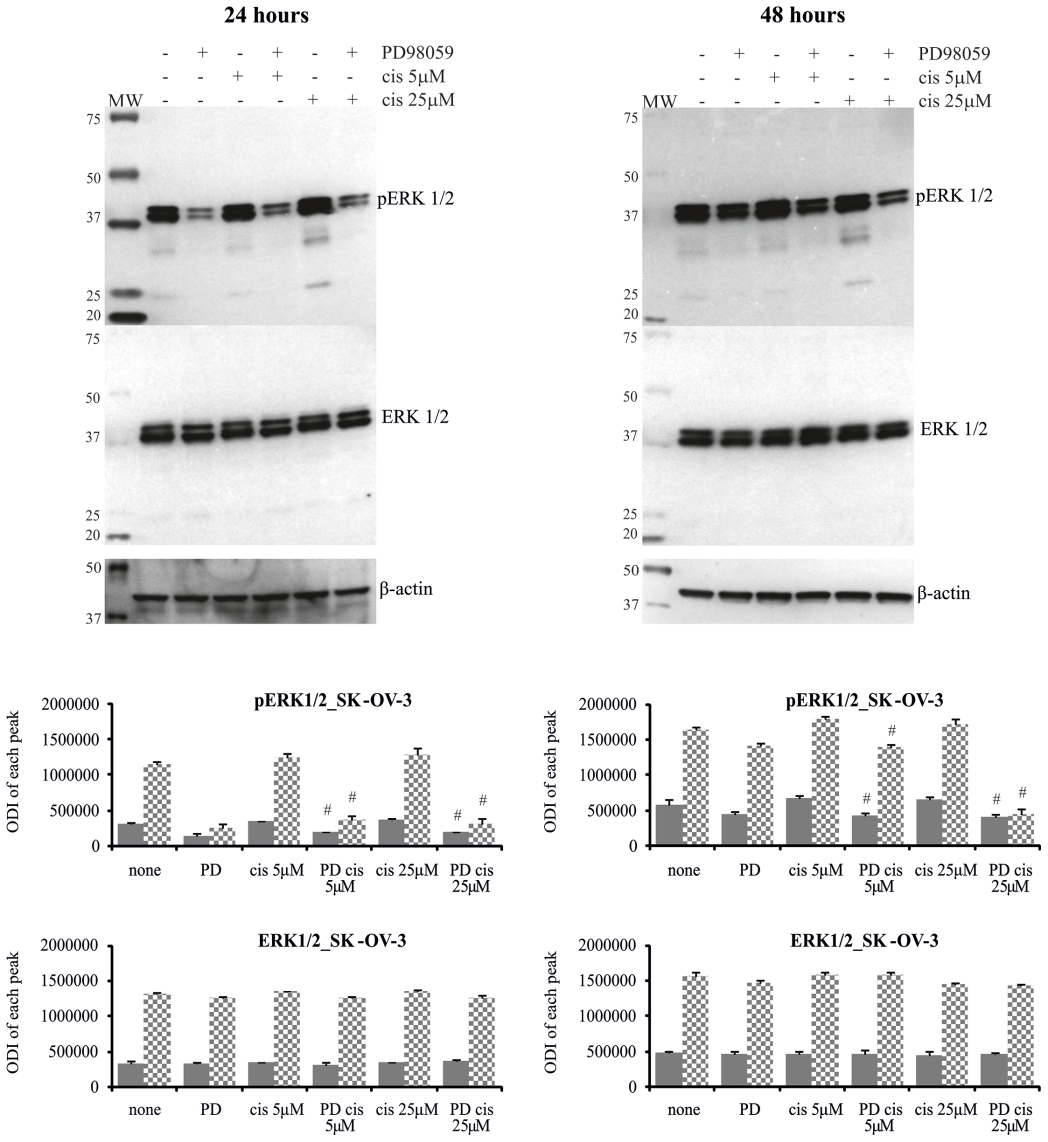
Phosphorylation of ERK1/2 in SK-OV-3 cells treated with MEK1/2 inhibitor and cisplatin Cells were first treated with 50 μM PD98059 for 1 hour and then cultured with cisplatin at the concentrations of 5 μM or 25 μM for 24 or 48 hours or left untreated. Representative immunoblots of the ERK1/2 protein level and their phosphorylation are presented. Bands were quantified by densitometric analysis and data are presented as the optical density intensity (ODI) of the area under each band’s peak ± SD from 5 independent experiments. ERK1 – gray column and ERK2 - checkered column. Statistically significant decrease: ^#^cisplatin + PD98059 vs. cisplatin, p≤0.05 (Wilcoxon’s signed-rank test).

The analysis of ERK1/2 phosphorylation in cells treated with cisplatin and inhibitor is presented on Figures 5 and 6. Only trace activity of ERK1/2 was observed in A2780 cells treated with PD98059 and cisplatin at low concentration for 24 or 48 hours. Low ERK1/2 activity was accompanied by higher sensitivity of cancer cells to the drug cytotoxicity, as mentioned above. We also observed that MEK1/2 inhibitor partially abrogated the cisplatin induced reduction in phosphorylation of ERK1/2 for sensitive cells exposed to 25 μM of this drug for 24 and 48 hours, resulting in a beneficial effect on cell survival in both time periods. PD98059 effectively reduced ERK1/2 phosphorylation in cisplatin treated A2780cis cells for 24 hours, which significantly enhanced cell death independently of the drug concentration. It is interesting that cytotoxicity of cisplatin for these cell treated with combination of PD98059 and 5μM of cisplatin versus treated with 25 μM of cisplatin alone is equal, while the phosphorylation status of ERK1/2 differ substantially. We also found that inhibitor fully ablated the activity of both kinases in A2780cis cells at 48 hours after dosing with 25 μM of cisplatin, but we did not find any link with cells survival. However, phosphorylation of ERK1/2 was still observed in A2780cis cells cultured with MEK1/2 inhibitor and cisplatin at a concentration of 5 μM for 48 hours, and the cells remained alive. It should be noted that although PD98059 strongly reduced ERK1/2 activity, it did not block the activity completely in the A2780cis cells cultured for 48 hours without any drug. PD9859 significantly but not completely decreased the phosphorylation of tested kinases in SK-OV-3 cells. However, it was difficult to find clear correlation between reduced ERK1/2 activity and survival of these cells treated with cisplatin.

### Effect of cisplatin and MEK1/2 inhibitor on the cell cycle of ovarian cancer cells

As presented in Table 1, most of the untreated A2780 cells (up to 67%) were in the G1 phase of the cell cycle. Cisplatin, at a concentration of 5 μM, strongly increased the numbers of A2780 cells in the S phase with both 24 and 48 hours of treatment. In parallel (as described above), the activity of ERK1/2 was enhanced, and most cells were alive. In the presence of ERK1/2 inhibitor and 5 μM of cisplatin, a slight but visible drop in the accumulation of cells in S phase was observed compared to a population cultured with drug alone in both time periods. Moreover, there was an enhanced number of cells in the G1 phase. Simultaneously, cisplatin had intensified cytotoxicity. Chemotherapeutic at a high concentration induced the accumulation of sensitive cells in the sub-G1 phase by more than 60% of the total cell population during the first 24 hours of exposure. As mentioned before, only slight activity of ERK1/2 was observed and most cells were dead at that time. Interestingly, PD98059 remarkably abolished the cisplatin-induced sub-G1 phase arrest of A2780 cells during 24 hours of treatment and most cells were in the S phase. At the same time, there was reversal of cisplatin cytotoxicity. Due to the high cytotoxicity of this drug at a high concentration for sensitive cells, it was not possible to measure the cell cycle with 48 hours of treatment. It is also interesting that PD98059 *per se* caused a slight (up to 10%) increase in A2780 cells accumulation in G1 phase compared to control.

**Table 1:** A2780 cells were first treated with 50 μM of PD98059 for 1 hour and next cultured with cisplatin at the concentrations of 5 μM or 25 μM for 24 or 48 hours or left untreated

Treatment of cells	24 hours			
	% < G1	% G1	% S	% G2
**none**	2.9 ± 1.4	59.7 ± 3.2	29.2 ± 3.0	7.1 ± 1.7
**PD98059**	1.8 ± 0.5	68.7 ± 1.9	23.7 ± 1.7	4.9 ± 0.8
**Cis 5μM**	2.1 ± 0.5	**17.1 ± 0.4^#^**	**73.1 ± 2.7^#^**	7.4 ± 2.1
**Cis 5μM + PD98059**	1.6 ± 0.8	29.9 ± 1.9^*^	62.3 ± 2.0^*^	6.4 ± 0.4
**Cis 25μM**	**68.8 ± 7.4^##^**	**16.9 ± 3.7^##^**	**12.7 ± 3.5^##^**	1.5 ± 0.9
**Cis 25μM + PD98059**	13.8 ± 8.1^**^	28.8 ± 5.1^**^	44.1 ± 4.1^**^	12.2 ± 1.5
**48 hours**				
**none**	2.1 ± 0.9	67.3 ± 1.7	24.3 ± 1.4	4.7 ± 0.7
**PD98059**	2.0 ± 0.9	69.3 ± 2.8	22.2 ± 1.7	5.9 ± 1.1
**Cis 5μM**	6.1 ± 2.0	**9.1 ± 0.5^#^**	**75.6 ± 0.9^#^**	8.6 ± 1.4
**Cis 5μM + PD98059**	4.5 ± 1.5	15.1 ± 1.6^*^	68.6 ± 1.8^*^	11.8 ± 2.0
**Cis 25μM**	n/a	n/a	n/a	n/a
**Cis 25μM + PD98059**	n/a	n/a	n/a	n/a

The cell cycle was determined by flow cytometry method. Data are presented as a percentage of cells being in sub-G1, G1, S and G2 phase ± SD from 3 independent experiments. Statistical significance (paired *t*-test): ^#^cis 5 μM vs. none, p≤0.0009; ^##^cis 25 μM vs. none, p≤0.002; ^*^cis 5 μM vs. cis 5 μM + PD98059, p≤0.02; ^**^cis 25 μM vs. cis 25 μM + PD98059, p≤0.03.

Untreated A2780cis cells were generally in the G1 phase (up to 65%) (Table 2). Cisplatin, at high concentration after both 24 and 48 hours of exposure, significantly enhanced the number of cells in the S phase and G2 phase. Simultaneously, as mentioned above, ERK1/2 were intensely phosphorylated and 26-40% percentage of cells died. A2780cis cells were proportionally distributed in G1 and S phase after 24 hours of treatment with cisplatin at concentration of 5 μM. Moreover, the percentage of cells in G2 phase was increased in comparison to untreated cells. This concentration of drug was not toxic for resistant cells and after 48 hours of exposure A2780cis cells were again found in G1 phase. When MEK1/2 inhibitor was added to cell culture before cisplatin, most of the cells were in the G1 phase, which is similar to untreated cells. This effect was independent of the chemotherapeutic drug concentration and culture time. However, it was highly difficult to identify a relationship between the viability of resistant cells exposure to PD98059 with cisplatin and the cycle phase. Moreover, inhibition of ERK1/2 activity (without a drug) resulted in a higher accumulation of A2780cis cells in the G1 phase (up to 80%).

**Table 2:** A2780cis cells were first treated with 50 μM of PD98059 for 1 hour and next cultured with cisplatin at the concentrations of 5 μM or 25 μM for 24 or 48 hours or left untreated

Treatment of cells	24 hours			
	% < G1	% G1	% S	% G2
**none**	3.0 ± 1.1	59.0 ± 1.6	32.0 ± 1.4	5.3 ± 1.3
**PD98059**	2.2 ± 0.4	79.1 ± 0.8	13.4 ± 1.7	4.7 ± 1.4
**Cis 5μM**	2.7 ± 1.4	**44.0 ± 2.1^#^**	**41.7 ± 2.4^#^**	11.1 1.5^#^
**Cis 5μM + PD98059**	2.5 ± 0.6	69.8 ± 3.0^*^	18.8 ± 2.1^*^	8.2 ± 0.4
**Cis 25μM**	4.4 ± 0.3	**29.6 ± 3.3^##^**	**57.0 ± 2.4^##^**	8.7 0.9^#^
**Cis 25μM + PD98059**	2.7 ± 0.4	50.9 ± 0.8^**^	37.1 ± 1.1^**^	9.1 ± 2.1
**48 hours**				
**none**	3.2 ± 1.3	64.4 ± 3.3	25.1 ± 3.0	6.6 ± 2.4
**PD98059**	1.5 ± 0.8	67.8 ± 5.0	22.4 ± 3.6	5.7 ± 1.0
**Cis 5μM**	3.1 ± 0.5	**53.1 ± 2.5^#^**	**27.1 ± 2.2^#^**	15.0 ± 1.8^#^
**Cis 5μM + PD98059**	1.5 ± 1.1	76.9 ± 2.3^*^	14.1 ± 1.5^*^	5.8 ± 0.9
**Cis 25μM**	9.0 ± 2.2	**20.1 ± 0.8^##^**	**59.4 ± 2.7^##^**	11.8 ± 1.5^#^
**Cis 25μM + PD98059**	3.7 ± 1.0	44.1 ± 3.4^**^	34.7 ± 2.1^**^	15.4 ± 1.3

The cell cycle was determined by flow cytometry method. Data are presented as a percentage of cells being in sub-G1, G1, S and G2 phase ± SD from 3 independent experiments. Statistical significance (paired *t*-test): ^#^cis 5 μM vs. none, p≤0.01; ^##^cis 25 μM vs. none, p≤0.007; ^*^cis 5 μM vs. cis 5 μM + PD98059, p≤0.004; ^**^cis 25 μM vs. cis 25 μM + PD98059, p≤0.02.

## DISCUSSION

Cisplatin has been reported to activate the ERK1/2 kinases in various ovarian cancer cell lines and that activation protects cells from chemotherapeutic-induced death [13, 14]. Conversely, studies have also demonstrated that cisplatin-induced ERK1/2 activation results in cell apoptosis [15, 16]. These controversies have not successfully been clarified yet. Therefore, the relationship between ERK1/2 activity and cisplatin cytotoxicity in association with the cell cycle in ovarian cancer cells resistant and sensitive to cisplatin was a subject of these studies.

Ovarian cancer cell lines used in our studies had constitutively phosphorylated ERK1/2, but it should be noted that cells resistant to cisplatin had these proteins phosphorylated to a significantly higher degree. Intensified basal activity of various kinases is wildly observed in ovarian cancer cells resistant to chemotherapeutic agents [17, 18]. The response of the tested cells to the cisplatin treatment was obviously dependent on three parameters, the drug dose, time of exposure and, most importantly, nature of the cancer cells. We showed that low cytotoxicity of cisplatin (independent of the concentration) against resistant A2780cis cells, during 24 hours of treatment, was highly related to this drug-induced enhanced activity of ERK1/2, measured as a phosphorylation state. Prolongation of the exposure time up to 48 hours sustained active ERK1/2 and most of the cells remained alive. Extremely high, basal level of phosphorylated ERK1/2 protected SK-OV-3 resistant cells form cytotoxicity of cisplatin, although this drug didn't affect the activity of kinases at any concentration used and time of treatment. Enhanced phosphorylation of ERK1/2 in various ovarian cancer cell lines, as a response to cisplatin exposure, was also reported by others [14, 19]. We also found that this chemotherapeutic, at a low dose, effectively increased the phosphorylation of both kinases (independent of the culture time) in the cisplatin-sensitive ovarian cancer cells (A2780), which could be the cause of its low cytotoxicity. Furthermore, our data showed that a high concentration of this drug was extremely toxic (after 24 and 48 hours in culture) for A2780 cells what was accompanied by a lack of intracellular ERK1/2. Excessive activation of ERK1/2 proteins results in their down-regulation, decreasing the anti-apoptotic properties and finally inducing cell death [7]. Moreover, cisplatin at high concentration over-activate ERK1/2 and induce the translocation of these protein from cytosol to nucleus resulting in its disappearance from cytosol [20]. Taken together, our data indicate that these proteins are important for protecting ovarian cancer cells from cisplatin-induced death; however, they do not keep cells alive when cisplatin at lethal concentration is applied. Others clearly described that ERK1/2 can have the dual role in cell survival and cell death [7].

Since the activation of ERK1/2 in general protects ovarian cancer cells from cisplatin cytotoxicity, we hypothesized that a pharmacological inhibitor (PD98059) that blocks these kinases, at the level of upstream kinases (MEK1/2), should increase the sensitivity of cells to the drug. However, the effect of PD98059 on the survival of A2780, A2780cis and SK-OV-3 cells in the presence of cisplatin was varied. We found that PD98059 significantly enhanced the cisplatin-induced cell death in A2780 cells at a low concentration of drug, which was accompanied by inhibitor-induced down-regulation of ERK1/2 activity. However, most A2780 cells remained alive. These findings indicate that active ERK1/2 proteins contribute pro-survival signals in ovarian cancer cells treated with a low dose of cisplatin, but they are not the sole kinases required for cell survival. On the other hand, the inhibitor had a beneficial effect on A2780 cell survival when a high concentration of cisplatin was used. Therefore the involvement of ERK1/2 in cell death or survival is complex [7] and it definitely depends on the concentration of the drug, since its high dose lead to disappearance of these kinases in A2780 cells. Moreover, it is essential to note that after 24 and 48 hours of A2780 cells exposure to PD98059 and cisplatin, at a concentration of 25μM, the phosphorylation of ERK1/2 signaling proteins was higher than in A2780 cells treated with cisplatin alone. Currently, we have no clear explanation for this phenomenon because MEK1/2 is the only known upstream activator of ERK1/2. However, others also observed a similar effect, showing that PD98059 blocked the cisplatin-induced apoptosis in A2780 cells [16], human myeloid leukemia cells [21] and human glioma cells [12]. The anti-apoptotic effect of MEK1/2 inhibitor was connected to its ability to reduce intracellular cisplatin accumulation *via* restoring the intracellular glutathione content [21]. Further explanation of the beneficial effect of PD98059 on the cell survival can be connected to Ras/Raf/MEK/ERK cross-talk with other signaling pathways, including PI3K/AKT/mTOR. It was reported that the Ras/Raf/MEK/ERK compensatory pathway was activated following the inhibition of PI3K/AKT/mTOR in human prostate cell lines [22].

Our research clearly showed that MEK1/2 inhibitor sensitizes A2780cis cells to cisplatin toxicity, independent of the concentration used, during the first 24 hours of treatment, which was accompanied by the blocked ERK1/2 phosphorylation. These findings support the statement that the signaling pathway based on ERK1/2 activity has pro-survival action. Similarly, it has been observed that a selective inhibitor of MEK1/2 (U0126), as well as siERK1/2, enhanced the OV433 ovarian cancer cell sensitivity to cisplatin, which were otherwise resistant to this drug, during 24 hours of treatment [14]. Above-mentioned data indicate that there is no straightforward way to explain the role of ERK/2 signaling proteins in the response of ovarian cancer cells to cisplatin. Prolonging the exposure time up to 48 hours showed no effect of PD98059 on cell survival in the presence of cisplatin, which confirms that there are multiple signaling pathways involved in the chemoresistance of ovarian cancer cells [3, 5]. It should be pointed out that PD9859 had no effect on the SK-OV-3 cells survival that once more shows dependence of ERK1/2 activity on various elements, including type of cells cancer cells and their intrinsic resistance to chemotherapeutics.

The implication of ERK1/2 in the cell cycle of cisplatin-treated cancer cells is even less established, showing the two-sided nature of these proteins. Data gained with a fibroblast model of cells indicated that ERK1/2 is required for cell cycle progression through the G1 phase. On the other hand, long-term and massive activation of these signaling proteins induced cell cycle arrest by the promotion p21cip1 protein (cyclic dependent kinase 1 inhibitor) accumulation [9, 23].

Our studies support the suggestion that cisplatin's influence on the ovarian cancer cell cycle was strictly related to the drug sensitivity and doses of these drugs. In general, cisplatin increased the number of resistant ovarian cancer cells in both the G1 and S phase, independently of the concentration, while ERK1/2 was highly active in these cells. MEK1/2 inhibitor enhanced the arrest of cisplatin-treated A2780cis cells in the G1 phase, preventing the cells from progressing into the S phase. Simultaneously, cell viability data indicate that PD98059 increased the cytotoxic effect of the drug. This observation is consistent with knowledge that lack of ERK1/2 blocks the entry of cells into S phase [9]. The relationship between the ERK1/2 activity and cell cycle was clearly visible in the sensitive ovarian cancer cells. We found that induction of ERK1/2 phosphorylation by cisplatin, at a low concentration, increased the number of cells in the S phase, and inhibition of these kinases *via* PD98509 shifted cell accumulation from the S phase to G1 phase. The cell cycle arrests in G1 enhanced the cytotoxic effect of a drug. Moreover, A2780 cells treated with cisplatin, at a high dose, were mostly in the sub-G1 phase and were characterized by a lack of intracellular ERK1/2 proteins. PD98059 reversed the cisplatin-induced cell cycle arrest in sub-G1 and A2780 cells that progressed to the G1 and S phase, which improved cell survival. It is accepted that DNA damage at low intensity activates ERK1/2, leading to cell cycle arrest, while extensive DNA damage induces over-activation of ERK and causes apoptosis [24]. The difference in the cell cycle progress between ovarian cancer cells resistant and sensitive to cisplatin may correlate with different checkpoint kinase activity. Sustained Chk1 activation was observed in the ovarian cancer cell line that was resistant to cisplatin – OVCA [25].

In summary, the relationship between the cytotoxicity of cisplatin, ERK1/2 activity and cell cycle progression primarily depends on the resistance of ovarian cancer cells to this drug and its concentration. In general, our data clearly indicate that ERK1/2 is activated by cisplatin, which keeps ovarian cancer cells alive and enhances their resistance to the cytotoxic action of this drug. However, intensified stimulation of these kinases by cisplatin at a high dose and/or with a longer exposition time leads to their down-regulation and cell death. Inhibition of ERK1/2 activity enhanced the cytotoxicity of cisplatin when used at a low concentration, while it had a beneficial effect on cell viability when the chemotherapeutic was used at a high concentration. Our finding also showed the importance of the ERK1/2 pathway in the cell cycle progression from the G1 to S phase in the ovarian cancer cells treated with cisplatin. MEK1/2 inhibitor enhanced the cytotoxicity of a drug by inducing G1 cell cycle arrest in both resistant and sensitive ovarian cancer cells. However, it should be underlined that this relationship was only observed when a low concentration of the chemotherapeutic was applied. The data indicate the biphasic nature of ERK1/2 and its complex role in the cells’ response to cisplatin, which should be considered during preparation of pre-clinical trials for pharmacological inhibitors of these kinases as supportive compounds for ovarian cancer treatment.

## MATERIALS AND METHODS

### Reagents and antibodies

Trypsin 0.05% EDTA solution, RPMI 1640 (Roswell Park Memorial Institute) medium with 2 mM L-glutamine and 1 mM sodium pyruvate, and Dulbecco's phosphate buffered saline (D-PBS) were purchased from Gibco (Inchinnan, Scotland). Fetal bovine serum (FBS) was obtained from PAN BIOTECH (Aidenbach, Germany). 3-(4,5-dimethylthiazol-2-yl)-2,5-diphenyltetrazolium bromide (MTT), cis-Diamineplatinum (II) dichloride, propidium iodide solution (PI), 2-(2-amino-3-metylofenylo)-4H-1-benzopiren-4-on (PD98059), Triton X-100, phenylmethylsulfonyl fluoride (PMSF), EGTA, EDTA, Tris, sodium dodecyl sulfate (SDS), *β*-mercaptoethanol, glycerol, Tween 20, bromophenyl blue, 2-propanol, Kodak Biomas Xar film, and penicillin/streptomycin solution were purchased from Sigma-Aldrich (St. Louis, MO, USA). SuperBlock Blocking Buffer in TBS, 10 x Tris-glycine-SDS buffer, halt protease and phosphatase inhibitor cocktail and ECL Western Blotting substrate kit were obtained from Thermo Scientific (Fremont, CA, USA). A DC protein assay kit, 10% SDS-PAGE mini-protean precast TGX gel, trans-blot turbo transfer pack PVDF and precision plus protein western C standard were obtained from BioRad (Hercules, CA, USA). The following antibodies: HRP-conjugated goat anti-rabbit IgG (H+L), HRP-conjugated goat anti-mouse IgG (H+L), rabbit polyclonal anti-MAPK, and rabbit polyclonal anti-phospho-ERK1+2 (Thr202/Tyr204) were purchased from Invitrogen (Carlsbad, CA, USA). Mouse IgG anti-*β* actin antibody was purchased from Sigma-Aldrich (St. Louis, MO, USA).

### Cell line culture and treatment

For these experiments, A2780 and A2780cis human ovarian cancer cell lines were purchased from Sigma-Aldrich and SK-OV-3 cells were purchased from the American Type Culture Collection. All cell lines are of epithelial origin, have an adherent growth type and were grown as a monolayer. Cells were cultured in RPMI 1640 medium containing 2 mM L-glutamine, 1 mM sodium pyruvate, 10% FBS, and 1% penicillin/streptomycin and they were passaged every 2-3 days by trypsinization (Trypsin 0.05% EDTA solution) for 10-15 minutes (37°C, 5% CO_2_). Cisplatin, at a concentration of 1 μM, was added to the A2780cis cell line every 2-3 passages to retain resistance to this drug. The viability of cells was assessed by trypan blue exclusion (>95%). All cells were free of Mycoplasma (tested with PlasmoTest^TM^ – Mycoplasma detection kit by Invivogen) and were harvested in the exponential growth phase before use.

Cancer cells suspended in culture medium (see above) were seeded in 96-well (MTT) or 24-well (PI assay, Immunobloting-ECL) plates at a concentration of 10^5^ cells/well or 10^6^ cells/well, respectively, and they were cultured for 24 hours to attach cells to the surface of the wells (37°C, 5% CO_2_). For cell cycle analysis, cancer cells were seeded in 24-well plates at densities of 2.5 and 3.0 × 10^5^ cells/well in culture medium for 24 hours. Next, culture medium was replaced and PD98059, at concentrations of 10, 25, 50, 100, 200, 500 μM, was added to cells for one hour or cells were left untreated. Afterwards, the dose of PD98059 was repeated in some samples, and cisplatin at various concentration (1, 5, 10, 25, 50, and 100 μM) was or was not to cells for 0.5, 1, 3, 6, 24, 48 or 72 hours (37°C, 5% CO_2_) (as indicated in the Results and Figures). Cells prepared in that way were then used for the MTT assay, PI exclusion, cell cycle assay or Immunoblotting-ECL test.

### MTT assay

After culturing cells with PD98059 and/or cisplatin, supernatants were removed and 100 μl of MTT solution (2 mg/ml) was added to each well. The cells were incubated for 3 hours at 37°C with a 5% CO_2_. Then, MTT was gently discarded and 200 μl of 2-propanol was added. The absorbance was measured on a Multiskan RC plate reader (Labsystem, Helsinki, Finland) with dual wavelengths of 595 nm and 630 nm using Genesis Lite software. The data were presented as the percentage of cytotoxicity, calculated according to the following formula:

$$\text{Cytotoxicity }\%=1-\frac{\text{optical density of sample}}{\text{optical density of control}}\times 100$$

### PI exclusion assay

After treatment, cells were harvested from plates, transferred into 5 ml tubes and washed twice with D-PBS supplemented with 1% of FBS. Then, cells were stained with PI (2 μg/ml D-PBS) for 30 minutes in the dark at room temperature. The intensity of PI fluorescence in each sample was measured the same day on a Becton Dickinson (San Jose, CA, USA) LSR II flow cytometer with BD FACS (fluorescence-activated cell sorting) Diva software. The fluorescence values were analyzed by WinMDI software. The data were presented as the percentage of dead cells.

### Immunoblotting-ECL

Cancer cell lines treated as mentioned above were harvested from 24-well plates, centrifuged (12000 x g, 2 minutes) and lysed with lysing buffer containing 1% Triton X 100, 20 mM Tris, 150 mM NaCl, 1 mM EDTA, 1 mM EGTA, 1 mM PMSF and 1% of halt protease and phosphatase inhibition cocktail for 30 minutes on ice. The lysates were stored at –70°C until analysis. The amount of protein in sample was measured using a DC Protein Assay kit. The cell lysates containing equal levels of proteins were run on a 10% SDS-PAGE mini-protean precast TGX gel and proteins were transferred to PVDF membranes using a Trans-Blot Turbo Transfer System (Bio-Rad) at 2.5A for 10 minutes. Afterward, the membranes were blocked with SuperBlock Blocking Buffer for 30 minutes and blotted with rabbit polyclonal anti-MAPK (1:1000), rabbit polyclonal anti-phospho-ERK1+2 [Thr202/Tyr20] (1:1000) or mouse IgG anti-βactin antibody (1:4000) (1 hour, room temperature). After washing the membranes (5 times in 2x TBS-Tween 20), they were incubated with secondary antibodies, HRP-conjugated goat anti-rabbit IgG (1:4000) or HRP-conjugated goat anti-mouse IgG (1:4000) (1 hour, room temperature), and again washed 5 times. Proteins were detected by the incubation of membranes with ECL Western Blotting Substrate. Proteins in blots underwent densitometric analysis using a FluoroChem MultiImage FC Cabinet (Alpha Innotech Corporation, San Leandro, CA, USA) and Alpha Ease FC software 3.1.2. The results are presented as the optical density intensity (ODI) of the area under each band's peak.

### Cell cycle analysis

After the culture of cells with MEK1/2 inhibitor and/or cisplatin, they were harvested and fixed in ice-cold 70% ethanol, stored for 24h in -20°C and were stained with staining solution (50 μg/ml propidium iodide and 200 μg/ml RNase A in phosphate-buffered saline) for 30 minutes in 37°C. Finally, the stained cells were analyzed using an LSRII flow cytometer (BD). Data were analyzed using FlowJo V10.

### Statistical analysis

Data are presented as the mean ± SD. The normality of the data distribution was verified by the D’Agostino - Pearsona K^2^ test. The data were analyzed with the paired *t*-test, unpaired *t*-test and Wilcoxon's signed-rank test using Statistica 12.0 for Windows. Statistical significance was defined as p ≤ 0.05.

## SUPPLEMENTARY MATERIALS FIGURE



## Abbreviations

ERK1/2: extracellular signal-regulated kinases 1 and 2
MEK1/2: mitogen activated protein kinase 1 and 2
MAPKs: mitogen-activated protein kinases
p21cip1 protein: cyclic dependent kinase 1 inhibitor
PI: propidium iodide
ODI: optical density intensity
EC_50_: half maximal effective concentration
