# Supplementary material for: Cisplatin-induced ERK1/2 activity promotes G1 to S phase progression which leads to chemoresistance of ovarian cancer cells
Source: Oncotarget. 2018 Apr 13;9(28):19847–60. doi: 10.18632/oncotarget.24884 (PMC5929431; doi:10.18632/oncotarget.24884)
Supplement: Supplementary file 1 [file oncotarget-09-19847-s001.pdf]

Cisplatin-induced ERK1/2 activity promotes G1 to S phase progression which leads to chemoresistance of ovarian cancer cells

SUPPLEMENTARY MATERIALS

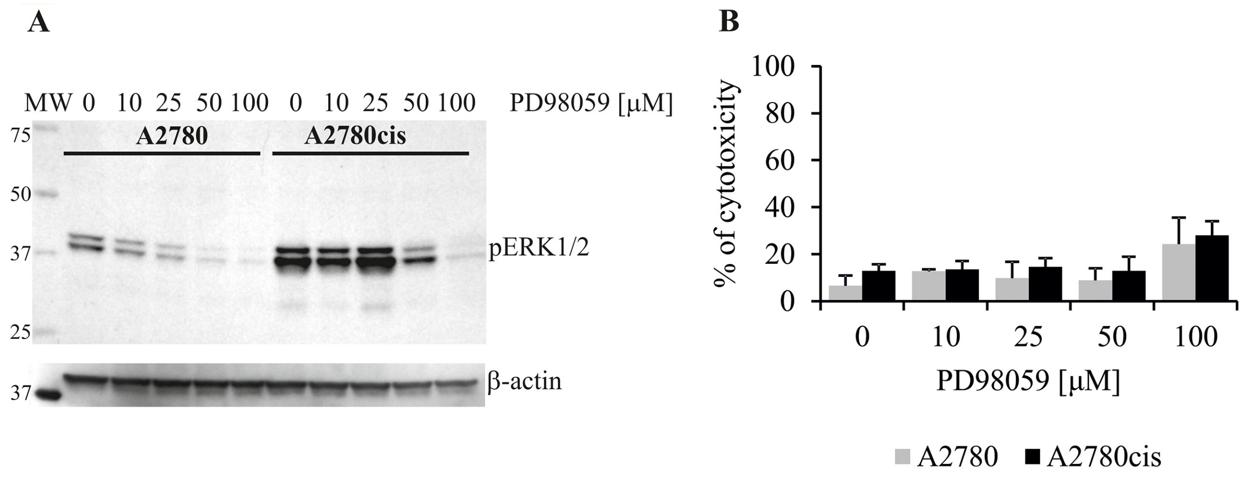

Supplementary Figure 1: Effect of PD98059 on ERK1/2 phosphorylation and cell viability. A2780 and A2780cis cells were treated with PD98059 at concentrations of 10, 20, 50 and 100 µM for 24 hours. **(A)** Representative immunoblots of the ERK1/2 protein phosphorylation levels are presented as one of 3 independent experiments. **(B)** The viability of the cells was determined with the MTT assay and data are presented as the percentage of cytotoxicity ± SD from 3 independent experiments.
